# Monthly-Term Associations Between Air Pollutants and Respiratory Morbidity in South Brazil 2013–2016: A Multi-City, Time-Series Analysis

**DOI:** 10.3390/ijerph16203787

**Published:** 2019-10-09

**Authors:** Dayana Milena Agudelo-Castañeda, Elba Calesso Teixeira, Larissa Alves, Julián Alfredo Fernández-Niño, Laura Andrea Rodríguez-Villamizar

**Affiliations:** 1 Department of Civil and Environmental Engineering, Universidad del Norte, 081007 Barranquilla, Colombia; 2 Postgraduate Program in Remote Sensing. Universidade Federal do Rio Grande do Sul, Av. Bento Gonçalves, RS 91501-970 Porto Alegre, Brazil; ecalessoteixeira@gmail.com; 3 Environmental Engineering program, Universidade Federal do Rio Grande do Sul, RS 91501-970 Porto Alegre, Brazil; issalvess@gmail.com; 4 Departamento de Salud Pública, Universidad del Norte, 081007 Barranquilla, Colombia; aninoj@uninorte.edu.co; 5 Departamento de Salud Pública, Universidad Industrial de Santander, 680002 Bucaramanga, Colombia

**Keywords:** air pollution, morbidity, adverse effects, epidemiology, Brazil

## Abstract

Most air pollution research conducted in Brazil has focused on assessing the daily-term effects of pollutants, but little is known about the health effects of air pollutants at an intermediate time term. The objective of this study was to determine the monthly-term association between air pollution and respiratory morbidity in five cities in South Brazil. An ecological time-series study was performed using the municipality as the unit of observation in five cities in South Brazil (Gravataí, Triunfo, Esteio, Canoas, and Charqueadas) between 2013 and 2016. Data for hospital admissions was obtained from the records of the Hospital Information Service. Air pollution data, including PM_10_, SO_2_, CO, NO_2_, and O_3_ (µg/m^3^) were obtained from the environmental government agency in Rio Grande do Sul State. Panel multivariable Poisson regression models were adjusted for monthly counts of respiratory hospitalizations. An increase of 10 μg/m^3^ in the monthly average concentration of PM_10_ was associated with an increase of respiratory hospitalizations in all age groups, with the maximum effect on the population aged between 16 and 59 years (IRR: Incidence rate ratio 2.04 (95% CI: Confidence interval = 1.97–2.12)). For NO_2_ and SO_2_, stronger intermediate-term effects were found in children aged between 6 and 15 years, while for O_3_ higher effects were found in children under 1 year. This is the first multi-city study conducted in South Brazil to account for intermediate-term effects of air pollutants on respiratory health.

## 1. Introduction

Large urban centers, characterized by growing industrial activity and specially vehicular traffic have led to an increase in all aspects of environmental pollution of which air pollution is a major issue considerably impacting the quality of life of urban populations [1]. Air pollutants and their health effects have become a major concern worldwide, especially in developing countries where the population is exposed to higher concentrations of pollutants and where there has been less success in dealing with air pollution control [2].

Several studies have shown an association between air pollution and morbidity as well as mortality for respiratory and cardiovascular diseases [3]. Atmospheric pollutants such as particulate matter (PM), nitrogen dioxide (NO_2_), carbon monoxide (CO), sulfur dioxide (SO_2_), and ozone (O_3_) are responsible for increasing mortality and morbidity, especially for respiratory and cardiovascular diseases [2,4,5,6]. Stronger evidence on the link between air pollutants and adverse health effects has been reported mainly for PM with an aerodynamic diameter of <10 μm (PM_10_) and fine PM (PM_2.5_) [7]. However, some studies have also found stronger health effects for gaseous pollutants (SO_2_ and NO_2_) [8,9]. Most of these studies documented associations between day-to-day air pollutant concentrations and variations in mortality, hospital admissions, and respiratory diseases. Moreover, effects of mixtures of air pollutants are complex and it is a matter in current environmental epidemiology research [10,11]. 

The effects of ambient temperature on morbidity are also a relevant public health issue. Every year, a large number of hospitalizations and deaths are associated with exposure to extreme ambient temperatures, especially during heat waves and cold spells [12]. These extreme weather variations related to morbidity occur more frequently among the elderly and children who are more vulnerable to weather changes [13]. Some studies have reported that as the climate changes, characterized by increases in the annual mean temperature and the frequency of heat waves in many areas, an increase in morbidity is expected in several regions including Europe, the Middle East, and North Africa. In addition, some authors [14] point to the association between cold spells and high morbidity for several countries, suggesting that extremely low temperatures can affect public health. Therefore, weather conditions, especially temperature and pluvial precipitation, which are closely linked to air pollutant concentrations, are important factors to be considered when assessing the health effects of air pollutant as they could be confounding variables.

The present study was carried out in Brazil, the largest country in South America, with a diverse urbanization level and weather conditions across cities, spanning from the tropical zone to the far end of South America. Most air pollution research conducted in Brazil has focused on assessing the daily-term effects of pollutants on mortality [15,16,17] and to a lesser extent in morbidity [18,19,20], but little is known about the health effects or air pollutants at an intermediate time term. There is a vast amount of information available so far regarding short-term and long-term exposure effects, however, the “harvesting” effect of air pollution on health effects, mainly in respiratory disease, have been described since 2000 but have been less frequently assessed in academic literature on air pollution and health effects [21]. This analysis focuses on the intermediate-term effects of particulate matter (PM_10_), SO_2_, CO, NO_2_, and O_3_, part of the group known as “criteria air pollutants” for their important role in air pollution mixtures and related health effects. Thus, the objective of this study was to determine the monthly-term association between air pollution and respiratory morbidity in five cities in South Brazil.

## 2. Materials and Methods

### 2.1. Study Area, Type of Study, Unit of Observation, and Population

An ecological time-series study was performed using the municipality as the unit of observation. The study population included all residents in 5 cities in South Brazil (Gravataí, Triunfo, Esteio, Canoas, and Charqueadas) between 2013 and 2016. The area selected for this study was the Metropolitan Area of Porto Alegre (MAPA) region, which is located at 29°30’S–30°30’S/50°25’W–51° 55’W, in the eastern part of Rio Grande do Sul state of Brazil containing a total area of 9800 km². This region corresponds to the main urbanized area of the state, which is located approximately 100 km from the Atlantic Ocean (east). This region has growing industrial activity and vehicular traffic, causing an intense increase in air pollutant concentrations. The study area experiences well-defined four seasons and its climate is strongly influenced by cold air masses migrating from the polar regions [22,23,24,25]. The wind direction shows marked seasonal variations; during summer and spring, the prevailing direction is east-southeast (E-SE) as opposed to west (W) in fall and northwest (NW) in winter. Further details of the study area are described by Landim et al. [26]. 

### 2.2. Data Sources

#### 2.2.1. Morbidity and Population Data 

Data for hospital admissions were obtained from the records of the Hospital Information Service of the Brazilian Unified Health System (DATASUS) [27] of 5 Brazilian cities: Gravataí, Triunfo, Esteio, Canoas, and Charqueadas, between 1 January 2013 and 31 December 2016. This database offers compiled monthly information including patient information such as hospital registration, hospitalization unit, discharge diagnosis, gender, age, residential address, and municipality of residence. Hospitalization discharge diagnoses were coded according to the *International Statistical Classification of Diseases and Related Health Problems*, 10th revision (ICD-10). We retrieved information for the present research for the following diseases grouped, including diseases of the Respiratory System (respiratory infections (J00–J06; J10–J18; J20–J22), asthma (J45–J46), and chronic obstructive pulmonary disease (J40–J44)). The information obtained from DATASUS was disaggregated by month-year and age groups.

The total population for each age group in each municipality was used as the “exposure” variable and was calculated based on national projections of total population per age group, year, and municipality. Since it can be reasonably assumed that there are practically no big changes in the population of a municipality within the same year, this adjustment is primarily for making the counts comparable among the age groups as well as among cities, given the differences in the total size of the susceptible population to develop the diseases. Additionally, the use of the variable allowed for estimate changes for incidence rates in the multivariable regression models. 

#### 2.2.2. Atmospheric Pollutants and Meteorological Data

Air pollution data, including PM_10_, SO_2_, CO, NO_2_, and O_3_ (µg/m^3^) were obtained from the database of air pollution data of the environmental government agency in Rio Grande do Sul state (Fundação Estadual de Proteção Ambiental Henrique Luiz Roessler FEPAM-RS). The location of the fixed stations were in 5 counties of RS as Gravataí (29°55’26”S/50°59’15”W), Esteio (29°51’33.5”S/51° 10’45.17”W), Canoas (29°52’59.83”S/51°8’41.44”W), Charqueadas (29°57'15,30”S/51°37’15.30”W), and Triunfo (29°56’27”S/51°41’34”W). Atmospheric pollutants were measured using validated data from automatic sequential analyzers. Atmospheric SO_2_ was measured using automatic sequential analyzers with the technique of ultraviolet fluorescence radiation. SO_2_ analyzers using the AF21M model of Environment S.A and AF22M model of Environment S.A. were used in the stations of Gravataí, Esteio, and Canoas. APSA-370 model SO_2_ analyzers of HORIBA Process and Environmental were used in stations Charqueadas and Triunfo. NO_2_ in the air was measured using a nitrogen oxide analyzer with a AC31M that uses the chemiluminescence method. O_3_ was measured by an O341M analyzer, using the photometry method of absorption of UV light. CO was measured by a carbon monoxide analyzer CO11M using the infrared absorption method. PM_10_ was measured by MP101M analyzer using the beta radiation method [22]. The monitoring of air pollutants was carried out continuously in µg/m^3^ at an average 15-min interval, in local time between 2013 and 2016, where the analyzers calculated every hour an average with 15-min mean concentrations. Thus, hourly means was recorded directly by the internal program of each analyzer. Then, daily means were only calculated when 75% of the data was available after the validation procedures were done by the environmental agency (FEPAM). All equipment was subjected to a rigid maintenance program, performing periodic calibrations. Since hospital admission data was available at a monthly basis and the aim of this study is to estimate intermediate effects, the daily concentrations for each pollutant were aggregated as monthly means of air pollutant concentrations in μg/m^3^. 

### 2.3. Statistical Analysis

For each municipality, monthly time-series databases using the total number of respiratory hospitalizations were consolidated alongside with monthly means concentrations of each air pollutant, monthly means of the values of weather variables, and the population by age group. Different graphic methods were used to perform an exploratory evaluation of the trajectory and seasonality of each time-series, including partial autocorrelation and cross-correlation plots. The time series of the total number of respiratory hospitalizations showed to fit well the Poisson distribution in the graphic methods and the equidispersion hypothesis was not consistently rejected by both the index of dispersion test (VIT) and the Bohning asymptotic test (*p* > 0.10). 

Panel multivariable Poisson regression models conditioned by year-month and city were built using a population average model form with an unstructured panel correlation in order to estimate the mean effects of the concentrations of each pollutant on the rates of hospitalizations across cities. As an alternative analysis, Poisson models were adjusted without time-stratification but by incorporating a term of seasonality using an amplitude (α) and phase component (φ), through the following expression:

(1) α∗cos(t+φ)= α1∗cos(t)−  α2∗sen(t)

The estimators obtained from both approaches were very similar. Results were presented for the models with the term of seasonality for their best performance according the Akaike criteria. All models were built independently by age group using as a response variable the number of hospitalizations for total respiratory diseases (*Y*), the monthly mean of air pollutants as an independent variable (X), and the total population of each municipality for that year and age (*τ*) as an offset variable. The logarithm of the rate *λ* was then modeled as E(*Y*)/τ, as a function of the independent variable *X*, so as to interpret the e^B^ associated with each *X* as an average percentage change in the rate of visits for each disease. The monthly average of pollutant concentrations were centered to the approximate whole value corresponding to 20% of the average of the time-series: NO_2_: 6 μg/m3, O_3_: 6 μg/m3, SO_2_: 1 μg/m^3^, and the PM_10_ and PM_2.5_ values were centered to 10 and 5 μg/m^3^, respectively. All models were adjusted by temperature and humidity. Model evaluation was conducted based on the distribution of residuals and goodness-of-fit tests. All of the analyses were done using STATA 14 (Stata Corporation, College Station, TX, USA).

## 3. Results

**Figure 1:**
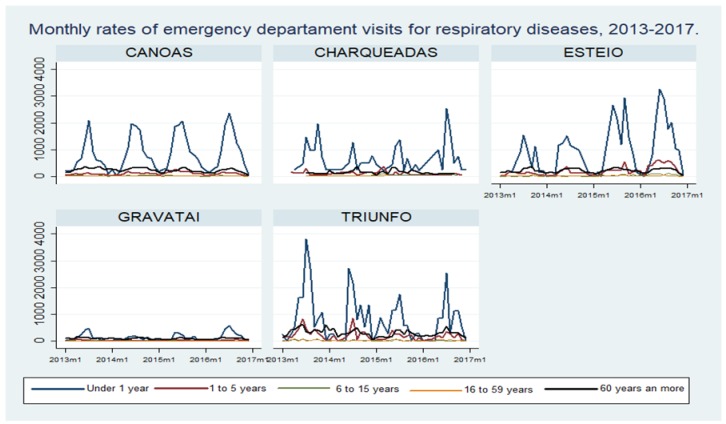
Monthly rates of hospitalizations per 100,000 inhabitants by municipality and age group, Rio Grande do Sul State, Brazil 2013–2016.

The total number of hospitalizations for respiratory diseases between 2013 and 2016 in the five municipalities was 21,708 (Table 1). The largest numbers of hospitalizations were registered in the municipality of Canoas and the lowest in Charqueadas. However, the historic rates per 100,000 inhabitants tend to be higher in Esteio y Triunfo. Figure 1 and Table 2 show the monthly rates of hospitalizations over the study period by municipality and age group, showing that children under 1 year had the highest rates of hospitalization, followed by older adults of 60 years and more.

The monthly average concentrations for PM_10_, NO_2_, SO_2_, CO, and O_3_ varied within the ranges for a minimum and maximum of 11.92–49.42 µg/m^3^, 1.16–48.65 µg/m^3^, 0.00–30.64 µg/m^3^, 0.00–0.101 µg/m^3^, and 2.36–125.18 µg/m^3^, respectively, for all studied municipalities (Table 3). Highest PM_10_ mean concentrations were observed in Canoas and Charqueadas, whereas CO and O_3_ were registered in Charqueadas, SO_2_ in Charqueadas and Triunfo, and NO_2_ in Canoas and Esteio.

Table 4 shows the results of the models by atmospheric pollutant and age group for respiratory diseases during the study period. An increase of 10 μg/m^3^ in the monthly average concentration of PM_10_ was associated with an increase of respiratory hospitalizations in all age groups with the maximum effect on the population between 16 and 59 years (IRR: Incidence rate ratio 2.04 (95% CI: Confidence interval = 1.97–2.12)), after controlling for the effect of meteorological variables and seasonality. For NO_2_ and SO_2_ stronger intermediate-term effects were found in the children population between 6 and 15 years, while for O_3_ higher effects were found in children under 1 year. 

## 4. Discussion 

This study found a clear association between monthly-term trend concentrations of PM_10_ and gases, and hospitalizations in all age groups in five cities in South Brazil. To our knowledge, this is the first study conducted in the area to account for intermediate-term effects of air pollutants on respiratory health. The results of the study suggest that an increase of about 20% of the mean concentrations of PM_10_, SO_2_, CO, NO_2_, and O_3_ have an immediate effect on increasing hospitalizations for respiratory conditions in all age groups, with a special impact of PM_10_ in the younger population and children under 5 years as well as in children between 6–15 years old for NO_2_.

There were differences in the concentrations and trends of air pollutant concentrations among cities that might explain the differences in health effects. Gravatai may be considered as a background site for atmospheric pollutants, as observed in previous research of the study area [26]. That study found that Gravatai is located upstream of the prevailing wind receiving a negligible anthropogenic load from local sources, while receiving regional/global pollution sources. The higher SO_2_ mean concentration observed in Charqueadas and Triunfo was expected because the predominant wind in the region is E and SE, thus these sites may be influenced by local pollution sources and the plume dispersion originated in the most urbanized and industrialized region of the state. Trends in the state were increasing, thus differing with other published trends in SO_2_ levels around the world, where concentrations are decreasing, possibly due to the contributions of thermoelectric power plants [26]. NO_2_ high levels presented in Canoas and Esteio were probably due to the influence of mobile sources of the BR-116 highway where diesel vehicles are responsible for most emissions of particulate matter and NO_x_, as well as other stationary sources such as the oil refinery and the steelworks [23,26,28]. PM_10_ mean levels were higher than WHO guidelines (25 µg/m^3^) for almost all municipalities, except Gravatai. Several studies conducted in the study area demonstrates that high levels were due to several emission sources such as mobile sources, industrial combustion, biomass burning, and particles resuspension [25,29,30]. The highest mean concentration for CO and O_3_ were registered in Charqueadas, probably because for O_3_ its concentrations are influenced by NO_x_ precursors and meteorological conditions (temperature and solar radiation, whereas CO is a major sink of hydroxyl radicals (OH)), which are mainly generated by O_3_ [23]. Moreover, in Charqueadas high O_3_ levels were influenced by the meteorological conditions and the atmospheric pollutants emitted from local sources. The passage of high thermal pressure systems accompanied by polar air masses, resulted in consecutive days of clear sky and mild winds, favoring the formation of O_3_ peaks that typically occur in heat waves combined with elevated global solar radiation and dry air in cities [31]. 

In our study, an increase of 10 μg/m^3^ in the monthly average concentration of PM_10_ was associated with an increase of respiratory hospitalizations in all age groups with the maximum effect on the population aged between 16 and 59 years (IRR 2.04 (95% CI = 1.97–2.12)). For NO_2_ and SO_2_, stronger intermediate-term effects were found in children aged between 6 and 15 years, while for O_3_ higher effects were found in children under 1 year. Most published time series studies related to air pollution and health effects in Brazil have been conducted in large cities such as São Paulo and identified adverse health effects in children and the elderly. Associations between mortality and levels of CO, SO_2_, and, to a lesser extent, PM_10_ were observed in São Paulo, for associations between mortality in elderly people and air pollution, using Generalized Additive Models (GAM) [32]. Other research has shown the impact of coarse-fraction exposure on hospital admissions among children due to respiratory diseases, where an increase of 5 μg/m^3^ in the coarse fraction concentration implied an increase in the relative risk of hospitalizations of up to 4.8% [33]. Another study was carried out using daily time-series from hospitalization and pollutant data in Vitoria, ES, Southeastern Brazil, from 2001 to 2006 showing that for an increment of 10 µg/m^3^ of the pollutants PM_10_, SO_2_, and O_3_, the percentage of relative risk (%RR) for hospitalizations due to total respiratory diseases increased by 9.6, 6.9, and 1.9, respectively [34]. In Cubatão, São Paulo State, Brazil, generalized additive Poisson regression models were used to model daily concentrations of PM_10_, SO_2_, and O_3_ and daily hospital admissions counts, showing the adverse effects of these pollutants on respiratory diseases for all ages [35]. Similar to our findings of the effects of NO_2_ in children, a study conducted in Ribeirão Preto was able to demonstrate that an increase of 10 ug/m^3^ in NO_2_ is associated with a RR of between 1.05 and 1.09 for having respiratory hospitalizations in children between 0 to 9 years [36]. 

Previous multi-city time-series studies conducted in Brazil that analyze the daily effects of air pollutants at the country level are scarce, due to the lack of monitoring data for most Brazilian cities. Freitas et al. [35] conducted a national study to select health impact indicators on air pollution and found that only six out of 27 states have municipalities with continuous monitoring air pollution systems between 2000 and 2008. The analyses conducted in 21 municipalities showed that hospitalizations for respiratory diseases in children under 5 years and in the general population were the best effect indicators for health surveillance. Miranda et al. [37] estimated the number of deaths associated with the excess exposure to PM_2.5_ for June 2007 to August 2008, based on experimental campaigns in six Brazilian state capitals for adults over 45 years, where São Paulo presented the worst results. Gouveia et al. [38] studied the impact of air pollution on respiratory hospitalization in nine municipalities of the metropolitan region of São Paulo and using meta-analysis found a combined RR of 1.011 for an increase in 10 ug/m^3^ of PM_10_. Recently, the relationship between the change in baseline and control scenarios of PM_2.5_ and excess of mortality was estimated using the BenMAP-CE program and the application of exposure-response functions in 24 Brazilian cities show that the maximum excess of deaths for all causes is produced in São Paulo [16].

Multi-city time series studies of the short-effect of air pollution on respiratory morbidity are scarce in other countries of South America. A recent multi-city time-series study of the effect of criteria air pollutants on emergency department visits for respiratory and cardiovascular diseases were conducted in Colombia [10]. Results were also stratified by age groups and similarly to this study, the highest effect of PM_10_ on respiratory conditions was found in children under 5 years while the highest effect of NO_2_ was found in children between 5 and 10 years. Effects observed for those pollutants on emergency department visits daily for four cities in Colombia were higher than the magnitude of effects of the same pollutants on monthly hospital admissions in these five cities in South Brazil. Although both studies cannot directly be compared due to differences in the type of morbidity indicator used (emergency versus hospitalizations) and the time indicator (daily versus monthly effect), the comparison suggests that the effects of air pollutants on health might be higher for acute (daily) exposure and mild respiratory conditions.

Studies on the long-term (chronic) effect of air pollutants on respiratory mortality and morbidity, using exposure models, have been conducted in North America, Asia, and European cities [7,39,40,41]. A recent synthesis of literature [42] showed that the relationship of long-term air pollution exposure with excess of mortality is better known than with morbidity and recommended that studies have to assess the progression of pollutant-related morbidity to mortality. In Brazil, a recent study used a land use regression model of NO_2_ as exposure for the long-term to assess the association between exposures to traffic-related air pollution and respiratory cancer incidence and mortality within São Paulo, Brazil [17]. Moreover, studies done with monthly-term data in Chinese cities observed the risk of lower respiratory diseases significantly correlated with concentration levels of SO_2_ and NO_2_ [43]. 

However, to our knowledge no studies on the long-term effect of air pollutants have been conducted in Brazil for respiratory morbidity. Therefore, this study adds important information on the estimations of intermediate-term health effects of criteria air pollutants in five cities in South Brazil that might be useful for having a better understanding of the progression of morbidity to mortality at an ecological level.

There are limitations in this type of time series studies that are important to discuss. First, time-series are ecologic studies that assume that air pollution exposure is homogeneous in all geographic units during the time unit. For this study it was assumed that the monthly mean concentrations of the air pollutants represent the actual population exposure and therefore a classic error might be introduced in the study for the aggregation of daily air pollution. However, a historical time-series analysis of air pollution have used aggregated data and offered the basis of the key messages of air pollution for public health. Additionally the availability of health data at monthly counts do not make it possible to have a smaller resolution for the analysis but offer the opportunity for exploring intermediate-term effects. Furthermore, this analysis assumes a single pollutant approach in which the interaction and combined effect of pollutants are not taken into account. Second, the quality of health data compiled by DATASUS might include misclassification bias that are inherent to the health administrations process but if present are non-differential across cities and months [36]. Third, of this study the number of data by city that did not allow us to stratify the effects by city so as to explore differences between them. For the reasons described before and the ecological approach used in this study, the results might not be extrapolated to an individual level.

There are also strengths of the study that are worth mentioning. Firstly, the measurement of air pollutants were obtained from certified equipment of a monitoring network that complies with international standards. Results of atmospheric pollutant concentrations and pattern have been well studied in the region [23,44] showing that despite the differences in air pollutant concentration levels, the effects are similar, including cities with low concentrations levels. Secondly, the statistical analysis using panel models with seasonality terms and population-level estimators let us obtain valid population average effects across the five cities. In addition, effects were obtained by pollutant and age group.

## 5. Conclusions 

This is the first multi-city study conducted in South Brazil to account for intermediate-term effects of air pollutants on respiratory health. The results of the study suggest that an increase of about 20% of the monthly mean concentrations of PM_10_, SO_2_, CO, NO_2_, and O_3_ had an immediate effect on increasing hospitalizations for respiratory conditions in all age groups with a special effect of PM_10_ on the young adult population and children under 5 years and of NO_2_ in children between 6–15 years.

## Figures and Tables

**Table 1:** Total counts and mean of monthly rates of hospitalizations per 100,000 inhabitants by year and municipality, Rio Grande do Sul State, Brazil 2013–2016.

City		2013	2014	2015	2016	Total
CANOAS	Count (sum)	3348	3691	3376	2843	13,258
	Rate (mean)	8.05	8.81	8.02	6.73	7.90
CHARQUEADAS	Count (sum)	73	113	190	91	467
	Rate (mean)	1.66	2.55	4.26	2.05	2.63
ESTEIO	Count (sum)	704	726	967	1071	3468
	Rate (mean)	6.87	7.03	9.46	10.47	8.46
GRAVATAI	Count (sum)	961	827	766	797	3351
	Rate (mean)	2.96	2.52	2.33	2.42	2.56
TRIUNFO	Count (sum)	384	284	243	253	1164
	Rate (mean)	12.14	8.97	7.71	8.04	9.22

**Table 2:** Total counts and mean of monthly rates of hospitalizations per 100,000 inhabitants by age group and municipality, Rio Grande do Sul State, Brazil 2013–2016.

		Age Group (years)				
City		Under 1	1 to 5	6 to 14	15 to 59	60 or More
CANOAS	Count (sum)	2244				
	Rate (mean)	86.23	11.6	3.9	3.28	36.06
CHARQUEADAS	Count (sum)	101	59	50	330	312
	Rate (mean)	68.95	11.87	6.6	3.97	23.91
ESTEIO	Count (sum)	518	450	281	872	1347
	Rate (mean)	93.56	20.38	4.29	3.37	37.98
GRAVATAI	Count (sum)	254	213	238	926	1720
	Rate (mean)	14.36	3.1	1.28	1.09	18.08
TRIUNFO	Count (sum)	137	148	33	280	566
	Rate (mean)	79.06	20.44	1.79	3.54	40.7

**Table 3:** Monthly average concentrations of atmospheric pollutants and meteorological parameters by municipality, Rio Grande do Sul State, Brazil 2013–2016.

Municipality	Variable	No. Months	Min	*P* 25	*P* 50	Mean	*P* 75	Max
**CANOAS**	PM_10_ (µg/m^3^)	48	14.05	24.34	29.03	29.44	34.93	47.77
	NO_2_ (µg/m^3^)	26	2.16	12.67	23.42	24.22	36.93	48.65
	SO_2_ (µg/m^3^)	36	0.13	0.75	1.38	4.28	8.84	16.98
	CO (µg/m^3^)	39	–	0.01	0.03	0.10	0.17	0.43
	O_3_ (µg/m^3^)	48	2.36	31.05	47.99	43.33	58.71	69.31
	Temperature (°C)	48	11.03	15.75	20.05	19.92	23.69	27.62
	Pressure (hPa)	48	1009.07	1012.56	1014.71	1015.06	1017.54	1022.50
	Relative humidity (%)	48	63.42	74.85	79.57	78.51	82.38	90.09
**CHARQUEADAS**	PM_10_ (µg/m^3^)	48	–	26.70	30.15	29.45	34.10	49.42
	NO_2_ (µg/m^3^)	48	5.87	7.89	10.49	10.93	13.15	19.06
	SO_2_ (µg/m^3^)	47	0.78	10.05	14.46	12.29	16.56	19.23
	CO (µg/m^3^)	48	0.15	0.21	0.47	0.53	0.79	1.01
	O_3_ (µg/m^3^)	48	19.54	32.93	44.47	51.39	58.67	125.18
	Temperature (°C)	48	10.66	15.71	20.15	19.64	23.64	26.23
	Pressure (hPa)	48	961.83	1010.94	1013.11	1012.06	1014.48	1020.55
	Relative humidity (%)	48	71.81	76.74	78.98	79.17	82.19	87.14
**ESTEIO**	PM_10_ (µg/m^3^)	23	14.54	18.40	19.82	23.00	26.86	43.75
	NO_2_ (µg/m^3^)	41	1.96	16.56	19.85	21.18	24.78	46.51
	SO_2_ (µg/m^3^)	36	0.89	2.90	4.48	5.91	7.74	19.96
	CO (µg/m^3^)	48	0.19	0.29	0.33	0.37	0.45	0.66
	O_3_ (µg/m^3^)	48	11.04	16.98	22.47	22.43	27.24	35.86
	Temperature (°C)	48	11.08	15.75	20.05	19.93	23.69	27.62
	Pressure (hPa)	48	1009.07	1012.56	1014.71	1015.06	1017.54	1022.50
	Relative humidity (%)	48	63.42	74.85	79.57	78.51	82.38	90.09
**GRAVATAI**	PM_10_ (µg/m^3^)	41	11.92	14.59	16.67	16.84	18.93	25.45
	NO_2_ (µg/m^3^)	22	1.17	2.54	5.03	6.77	10.77	17.32
	SO_2_ (µg/m^3^)	31	0.00	0.08	0.25	0.43	0.41	4.78
	CO (µg/m^3^)	39	0.00	0.13	0.20	0.21	0.29	0.59
	O_3_ (µg/m^3^)	22	12.43	18.46	28.42	26.56	35.17	38.35
	Temperature (°C)	48	11.44	16.26	20.56	19.95	23.74	26.49
	Pressure (hPa)	48	1004.67	1007.18	1009.70	1009.88	1012.06	1016.86
	Relative humidity (%)	48	68.27	74.32	77.37	77.67	81.01	87.39
**TRIUNFO**	PM_10_ (µg/m^3^)	–	.	.	.	.	.	.
	NO_2_ (µg/m^3^)	48	3.44	5.23	6.29	6.62	7.85	10.82
	SO_2_ (µg/m^3^)	44	1.16	9.85	13.08	13.37	17.32	30.64
	CO (µg/m^3^)	–	.	.	.	.	.	.
	O_3_ (µg/m^3^)	–	.	.	.	.	.	.
	Temperature (°C)	48	10.66	15.71	20.15	19.64	23.64	26.23
	Pressure (hPa)	48	961.83	1010.94	1013.11	1012.06	1014.48	1020.55
	Relative humidity (%)	48	71.81	76.74	78.98	79.17	82.19	87.14

**Table 4:** Results using Poisson Models for panel effects with population averages by atmospheric pollutant and age group for respiratory diseases, 2013–2016 *.

Air Pollutant	Age Group																			
	<1 year				<1–5 Years				6–15 Years				16–59 Years				>60 Years and More			
	IRR	CI 95%		*p*	IRR	CI 95%		*p*	IRR	CI 95%		*p*	IRR	CI 95%		*p*	IRR	CI 95%		*p*
**PM_10_**	1.33	1.28	1.38	<0.01	1.63	1.51	1.79	<0.01	1.37	1.28	1.48	<0.01	2.04	1.97	2.12	<0.01	1.08	1.07	1.11	<0.01
**NO_2_**	1.11	1.08	1.14	<0.01	0.89	0.88	0.91	<0.01	1.14	1.11	1.17	<0.01	0.97	0.95	1.01	0.1	1.04	1.02	1.07	<0.01
**SO_2_**	1.05	1.05	1.07	<0.01	1.05	1.04	1.06	<0.01	1.1	1.08	1.11	<0.01	0.97	0.94	1.01	0.1	1.01	1	1.01	0.01
**O_3_**	1.1	1.08	1.11	<0.01	1.06	1.03	1.08	<0.01	1.04	1.03	1.06	<0.01	1.03	1.02	1.05	<0.01	1.02	1.01	1.03	<0.01

* Adjusted for temperature, humidity and seasonality. IRR: Incidence rate ratio; CI: Confidence interval; *p*: *p*-value.
